# Pancreatic hydatid cyst with a characteristic “water-lily” sign

**DOI:** 10.1093/bjrcr/uaag042

**Published:** 2026-10-13

**Authors:** Aminreza Abkhoo, Niloufar Ayoobi, Seyed Mohammad Amin Dashti, Faeze Salahshour

**Affiliations:** Department of Radiology, School of Medicine, Advanced Diagnostic and Interventional Radiology Research Center (ADIR), Imam Khomeini Hospital, Tehran University of Medical Sciences, Tehran 14194, Iran; Department of Radiology, School of Medicine, Advanced Diagnostic and Interventional Radiology Research Center (ADIR), Imam Khomeini Hospital, Tehran University of Medical Sciences, Tehran 14194, Iran; School of Medicine, Tehran University of Medical Sciences (TUMS), Poursina Street, Qods Street, Enghelab Street, Tehran 1461884513, Iran; Department of Radiology, School of Medicine, Advanced Diagnostic and Interventional Radiology Research Center (ADIR), Imam Khomeini Hospital, Tehran University of Medical Sciences, Tehran 14194, Iran

**Keywords:** hydatid disease, echinococcosis, pancreas, pancreatic cyst, water-lily sign, magnetic resonance imaging, magnetic resonance cholangiopancreatography, endoscopic ultrasound-guided fine needle aspiration, albendazole

## Abstract

**Background:**

Cystic echinococcosis rarely involves the pancreas and may mimic common cystic neoplasms. Recognition of characteristic imaging signs is central to diagnosis; the “water-lily” sign (floating detached endocyst) is a rare and highly characteristic finding in favor of transitional-stage hydatid cyst and, when recognized, can prevent inappropriate cyst puncture and guide stage- and anatomy-based management.

**Case:**

A 34-year-old woman from Iran, an endemic region for cystic echinococcosis, presented with intermittent epigastric/right upper quadrant pain. At an outside institution, endoscopic ultrasound-guided fine-needle aspiration had been performed before referral; cyst-fluid amylase was 20 U/L and carcinoembryonic antigen was not detected, arguing against pseudocyst and mucinous neoplasm. Ultrasound and MRI/MRCP performed after EUS showed a unilocular pancreatic head cyst with a floating internal membrane (“water-lily”/“serpent” appearance) and mild upstream pancreatic duct dilatation. Our multidisciplinary team favored pancreatic cystic echinococcosis. The patient underwent surgical cyst excision with avoidance of scolicidal instillation into the biliary tree. Albendazole (400 mg twice daily, ∼ 10-15 mg/kg/day) was given perioperatively and continued for 6 months. Pathology confirmed laminated membranes and protoscolices. She remains asymptomatic on surveillance MRI without recurrence.

**Conclusion:**

In an endemic region, a pancreatic cyst with a floating membrane and low cyst-fluid amylase should raise suspicion for cystic echinococcosis. Anchoring the diagnosis to WHO staging and resisting routine EUS-FNA can avert spillage and caustic cholangiopathy from scolicidal agents. Radiologists play the key role in making this distinction.

## Introduction

Cystic echinococcosis (CE) is a chronic parasitic disease caused by Echinococcus granulosus.^1^ While the liver and lungs account for most cases, pancreatic involvement is uncommon yet well described. Because pancreatic CE frequently imitates mucinous cystic neoplasms (MCNs) or pseudocysts, precise radiologic characterization is essential.^2^^,^^3^ The WHO-IWGE staging system links imaging appearances to activity and guides therapy.^1^ We report a pancreatic-head CE with a conspicuous floating membrane (“water-lily” sign), review the differential against pancreatic cystic neoplasia, and align management with contemporary WHO guidance. The educational value of this case lies in recognizing the characteristic water-lily sign in the rare pancreatic location, particularly when it becomes apparent only after prior invasive sampling.

## Case presentation

A 34-year-old woman from Iran, an endemic region for cystic echinococcosis, presented with intermittent epigastric pain radiating to the back and occasional nausea. Physical examination showed mild epigastric tenderness without jaundice. Routine blood tests, including liver enzymes and inflammatory markers, were within reference ranges. Tumor markers—serum carcinoembryonic antigen (CEA) and carbohydrate antigen 19-9 (CA 19-9)—were not elevated.

**Figure 1:**
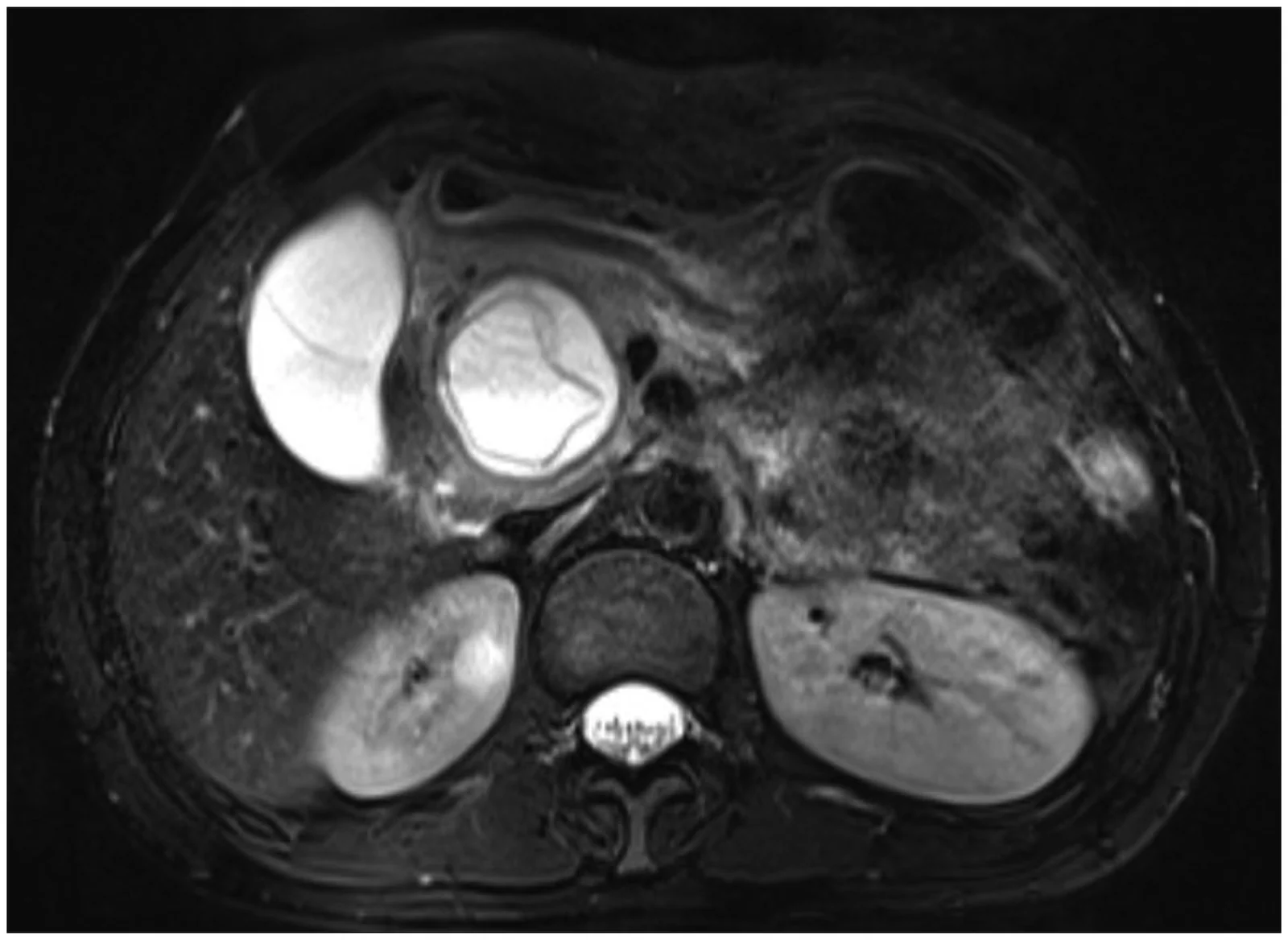
Axial fat-suppressed T2-weighted magnetic resonance image demonstrates a thin-walled, markedly T2-hyperintense unilocular cyst in the pancreatic head containing an undulating detached internal membrane (arrows), producing the characteristic “water-lily” sign.

Abdominal ultrasound identified a well-circumscribed anechoic cyst in the pancreatic head with fine internal echoes. Before referral to our center, the patient underwent endoscopic ultrasound-guided fine-needle aspiration (EUS-FNA) at an outside institution. The cyst appeared simple on EUS exam. Cyst-fluid analysis showed an amylase concentration of 20 U/L, while CEA was not detected. No immediate complication, including anaphylaxis, was recorded after the procedure. The low cyst-fluid amylase concentration argued against a pancreatic pseudocyst, while undetectable cyst-fluid CEA made a mucinous cystic lesion less likely; however, these markers were not interpreted in isolation.^4^^,^^5^

**Figure 2:**
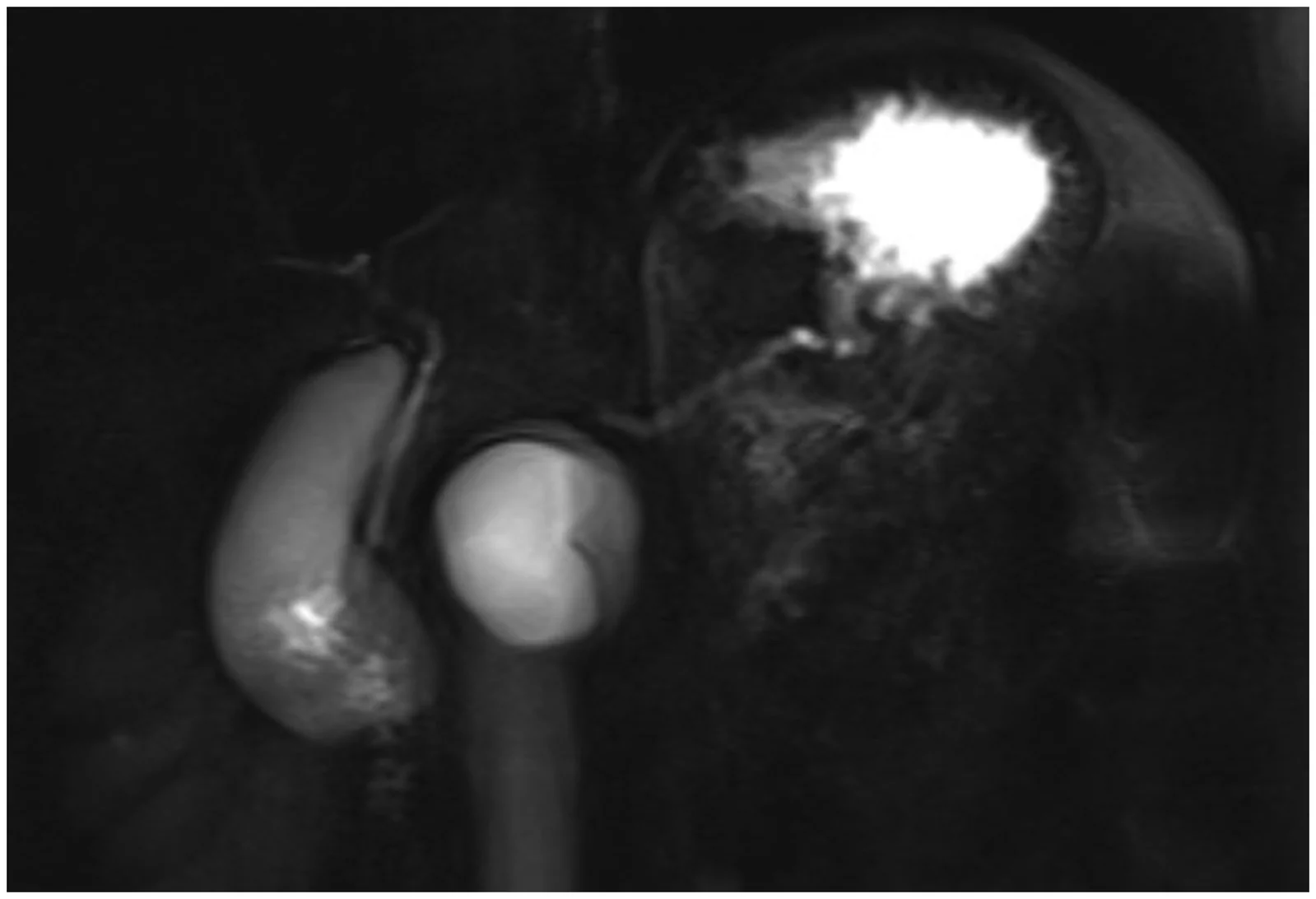
Magnetic resonance cholangiopancreatography image demonstrates the pancreatic-head cyst with mild upstream main pancreatic duct dilatation. No definite communication between the cyst and the pancreatic duct is identified.

On day 7, 4 days after EUS-FNA performed on day 3, non-contrast magnetic resonance cholangiopancreatography (MRCP) demonstrated a thin-walled, T2-hyperintense unilocular cyst measuring approximately 3-4 cm in the pancreatic head, containing a floating corrugated internal membrane and associated with mild upstream pancreatic duct dilatation. No additional abdominal or thoracic hydatid cysts were detected.

**Figure 3:**
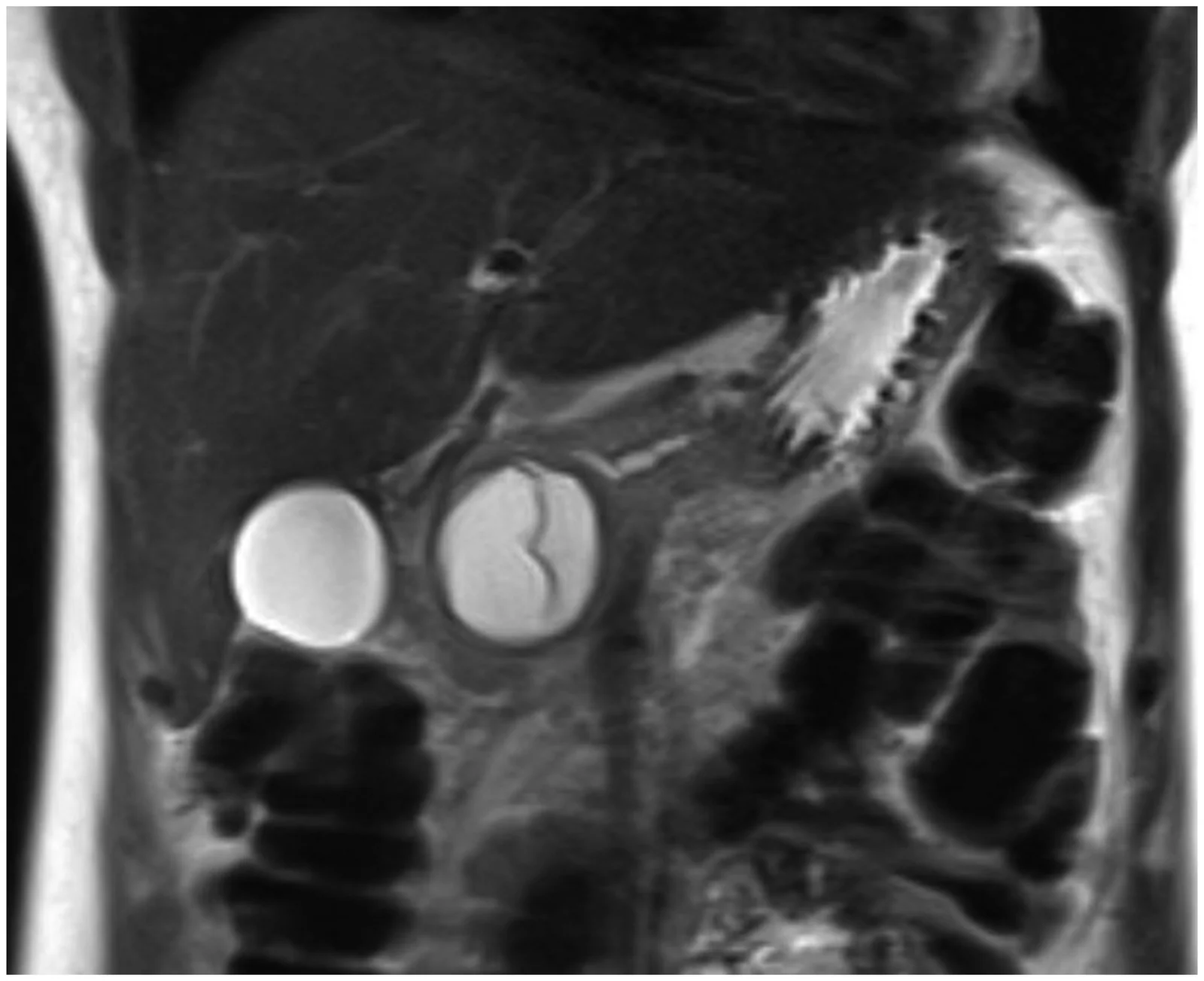
Coronal T2-weighted magnetic resonance image provides orthogonal demonstration of the detached corrugated membrane within the pancreatic-head cyst, confirming the “water-lily” appearance. Mild upstream main pancreatic duct dilatation is also present.

Serum anti-*Echinococcus* IgG measured by ELISA was negative. Given the floating membrane, endemic background, early obstructive features, rarity of isolated pancreatic hydatid cyst, and residual uncertainty about cystic neoplasia, the case was discussed at a multidisciplinary team (MDT) meeting. Pancreatic CE, most consistent with WHO-IWGE CE3a,^1^ was favored, and surgical treatment was selected. Albendazole (400 mg twice daily) was initiated perioperatively.

At surgery, a unilocular cyst was enucleated from the pancreatic head without intentional instillation of scolicidal agents into the biliary tree. Histopathology demonstrated laminated membranes and protoscolices/hooklets, confirming hydatid disease. Albendazole was continued for a 6-month course with monthly liver-function monitoring. Follow-up MRI at 6 and 12 months showed no recurrence; the patient remains asymptomatic.

## Imaging findings

Ultrasound demonstrated a unilocular cyst in the pancreatic head with fine internal echoes. The available examination consisted of non-contrast MRCP sequences, which demonstrated a thin-walled, markedly T2-hyperintense lesion containing a frond-like, undulating floating membrane, consistent with the “water-lily” sign of a WHO-IWGE CE3a hydatid cyst (Figure 1). Dedicated pancreatic T1-weighted and contrast-enhanced sequences were not available; therefore, the T1 signal of the cyst and upstream pancreatic parenchyma and the enhancement pattern could not be assessed. This membrane was detected on MRI performed after EUS-FNA and had not been described on the pre-referral EUS examination. The membrane may have detached during spontaneous cyst evolution, may have been overlooked during the initial EUS examination, or may represent a post-procedural change following aspiration; the available examinations do not allow these possibilities to be distinguished with certainty. MRCP depicted mild upstream pancreatic-duct dilatation and no definite cyst-duct communication (Figures 2 and 3). No hepatic, splenic, or pulmonary hydatid cysts were seen. Based on WHO-IWGE staging, these features are most compatible with a transitional CE3a cyst.^1^^,^^6^^,^^7^

## Diagnostic assessment and differential diagnosis

In an adult woman with a macrocystic pancreatic lesion, the principal differentials were MCN, branch-duct intraductal papillary mucinous neoplasm (IPMN), serous cystadenoma (SCA), pancreatic pseudocyst, solid pseudopapillary epithelial neoplasm, and cystic neuroendocrine tumor. The conspicuous floating membrane was highly characteristic of hydatid disease. Cyst-fluid analysis supported a non-pseudocyst, non-mucinous process (low amylase; CEA not elevated). A focused differential matrix is provided in Table 1.

**Table 1:** Differential diagnosis of a pancreatic cyst with internal debris/membranes.

Entity	Typical imaging pattern	Cyst-fluid CEA	Cyst-fluid amylase	Usual demographics/site	Relevance to this case
**MCN**	Unilocular/macrocystic; thick wall; no duct communication	Often high; ∼192 ng/mL threshold commonly used for mucinous	Low	Women; 40-60 yrs; body/tail	Head location; undetectable cyst-fluid CEA and the floating membrane argue against
**Branch-duct IPMN**	Multiloculated; communicates with duct; mural nodules may occur	Often high	High (duct communication)	Both sexes; older; any site	No definite ductal communication; no mucin extrusion
**SCA**	Microcystic “honeycomb”; central scar/calcification	Low	Low	Women; >50 yrs; no fixed pancreatic site	Imaging not microcystic; internal membrane atypical
**Pseudocyst**	Unilocular; peripancreatic; history of pancreatitis	Low	High (usually thousands), due to duct leak	Any; post-pancreatitis	Cyst-fluid amylase of 20 U/L and no history of pancreatitis argue strongly against
**Cystic neuroendocrine tumor**	Hypervascular solid-cystic; may enhance periphery	Variable	Variable/low	Any; no fixed pancreatic site	No enhancing solid component; serology/imaging favor CE
**Solid pseudopapillary neoplasm**	Well-circumscribed encapsulated solid-cystic mass, frequently with hemorrhagic components; solid tissue may enhance	Usually low or undetectable	Usually low (<250 U/L)	Young women; commonly body or tail	No definite solid component on the submitted T2 images; a detached membrane favors echinococcosis
**Pancreatic cystic echinococcosis, CE3a**	Unilocular cyst with detached floating membrane producing the water-lily sign; a low-signal peripheral rim may be present	No established diagnostic CEA profile	No established diagnostic amylase profile	Endemic region; pancreatic involvement is rare	The floating membrane is the strongest distinguishing feature in this case

Abbreviations: CE = cystic echinococcosis, CEA = carcinoembryonic antigen, IPMN = intraductal papillary mucinous neoplasm, MCN = mucinous cystic neoplasm, SCA = serous cystadenoma, SPN = solid pseudopapillary neoplasm, Units: ng/mL = nanograms per milliliter, U/L = units per liter.

## Therapeutic intervention

Because of early obstructive features and persistent, even low concern for a cystic neoplasm, the MDT recommended surgical excision. The absence of an immediate anaphylactic reaction after the pre-referral EUS-FNA was fortunate; however, this case reinforces that puncture should be avoided when hydatid disease is suspected on imaging.^1^^,^^2^ Perioperative albendazole was started and continued post-operatively for 6 months. Intra-operative scolicidal instillation into the cyst or biliary tree was deliberately avoided to mitigate the recognized risk of caustic sclerosing cholangitis should occult cystobiliary communication be present. Pathology confirmed hydatid disease.

## Follow-up and outcomes

The patient completed 6 months of albendazole (400 mg twice daily; ∼ 10-15 mg/kg/day) without adverse effects. Monthly liver tests remained within acceptable limits. MRI at 6 and 12 months showed no residual or recurrent disease. She remains asymptomatic and has returned to usual activities (Table 2).

**Table 2:** Timeline of clinical course.

Time	Event	Notes/key data
**Day 0**	Initial presentation	Intermittent epigastric/RUQ pain; exam unremarkable; labs within reference ranges.
**Day 3**	EUS-FNA in another center	Cyst-fluid amylase 20 U/L; CEA not detected; no immediate complication recorded.
**Day 7**	US + MRI/MRCP at our center	Unilocular pancreatic-head cyst with floating internal membrane (“water-lily”/“serpent” sign); mild upstream pancreatic duct dilatation.
**Day 13**	MDT discussion	Favor pancreatic CE (WHO CE3a) over mucinous neoplasm; plan surgery; start albendazole peri-operatively.
**Day 25**	Surgical cyst excision	Enucleation; avoidance of scolicidal instillation into the biliary system.
**Months 1-6**	Albendazole continuation	400 mg twice daily (≈10-15 mg/kg/day); monthly LFTs; no adverse events.
**Months 6 and 12**	Surveillance MRI	No recurrence; patient asymptomatic.

Abbreviations: CE = cystic echinococcosis, CE3a = transitional cyst stage characterized by detached membranes, CEA = carcinoembryonic antigen, EUS-FNA = endoscopic ultrasound-guided fine-needle aspiration, LFTs = liver function tests, MDT = multidisciplinary team, MRCP = magnetic resonance cholangiopancreatography, MRI = magnetic resonance imaging, RUQ = right upper quadrant, Unit: U/L = units per liter, US = ultrasound, WHO = World Health Organization.

## Discussion

Pancreatic cystic echinococcosis is uncommon, but it has been described in both surgical and radiologic series. Its imaging appearance may overlap with that of pancreatic cystic neoplasms, particularly mucinous lesions.^2^^,^^3^ The presence of detached laminated membranes within the cyst produces the classic “water-lily” sign, also described as a “serpent” or “whirl” appearance.^5^^,^^7^ In a patient from an endemic region, this finding strongly supports the diagnosis of hydatid disease.

Similar diagnostic difficulty has been described in previous pancreatic cases. Wu et al identified 33 primary pancreatic hydatid cysts reported between 2011 and 2021, including 14 lesions in the pancreatic head; 8 of 19 cases with reported IgG serology were seronegative. Kothiya et al documented evolution of a simple-appearing pancreatic hydatid cyst to a CE3a cyst with floating membranes, supporting spontaneous membrane detachment as one possible explanation. In contrast, Soin et al demonstrated floating membranes directly on EUS, indicating that this feature may sometimes be recognized before aspiration.^3^^,^^8^^,^^9^

Cyst-fluid analysis may help when the diagnosis remains uncertain. A cyst-fluid amylase level below 250 U/L argues against a pseudocyst by suggesting lack of communication with the pancreatic duct. Cyst-fluid CEA is more useful for differentiating mucinous from non-mucinous cysts than for predicting malignancy.^4^

Management of CE is generally guided by cyst stage according to the WHO-IWGE classification. For CE1 and CE3a cysts in accessible organs, albendazole alone or in combination with image-guided treatment may be appropriate. In pancreatic head lesions, however, surgery is often selected when there is biliary or pancreatic duct obstruction, diagnostic uncertainty, or concern for a cystic neoplasm.^1^^,^^2^ Routine EUS-FNA should be avoided when characteristic hydatid features are already present, because puncture carries a risk of spillage and anaphylaxis and is unlikely to alter management. When intervention is performed, scolicidal agents should be prevented from entering the biliary tree because of the risk of chemical cholangiopathy.^10^

Clinician’s reflection: in our multidisciplinary discussion, the floating membrane on MRCP was the key finding that shifted the diagnosis away from a pancreatic cystic tumor and toward pancreatic CE. For radiologists, the practical message is straightforward: in an endemic setting, a cyst with a floating internal membrane should first raise the possibility of hydatid disease, and invasive sampling should be reconsidered before it is performed.

## Learning points for radiologists and MDTs

- A floating internal membrane within a pancreatic cyst, corresponding to the “water-lily” or “serpent” sign, is a characteristic imaging feature of cystic echinococcosis and should be described clearly in the report.
- Low cyst-fluid amylase, particularly below 250 U/L, argues against a pancreatic pseudocyst. Cyst-fluid CEA is useful mainly for distinguishing mucinous from non-mucinous cysts, rather than for determining malignancy.
- Imaging descriptions should be linked to the WHO-IWGE stage of cystic echinococcosis, as management depends on cyst stage and anatomical site. Surgery may be appropriate for pancreatic head lesions causing obstruction or when a cystic neoplasm cannot be confidently excluded.
- Routine EUS-FNA should be avoided when imaging already shows characteristic features of hydatid disease. If intervention is performed, measures should be taken to minimize spillage and to prevent scolicidal agents from entering the biliary tree.

## Data Availability

Data sharing is not applicable to this case report because no datasets were generated or analyzed beyond the clinical information reported in the manuscript.

## Patient perspective

The patient reported that having a clear name for the condition and a plan that avoided repeated aspirations helped her anxiety; she was relieved to return to normal activities without recurrence.
